# Development of a simple clinical score to predict early neurological improvement after mechanical thrombectomy: a single-centre cohort study

**DOI:** 10.1186/s12883-026-04927-0

**Published:** 2026-05-09

**Authors:** Chenyang Zhao, Xihua Li, Yi Han, Tao Zhou, Xuefei Ren, Yaxuan Sun

**Affiliations:** Department of Neurology, Shanxi Provincial People’s Hospital, Shanxi Medical University, Heping Campus, Jiancaoping District, Taiyuan, Shanxi 030012 China

**Keywords:** Stroke, Thrombectomy, Eearly neurological improvement, Prediction model, Risk score, Nomogram

## Abstract

**Background:**

Early neurological improvement (ENI) after endovascular thrombectomy (EVT) is a clinically relevant early outcome and has been associated with subsequent functional recovery. However, simple bedside approaches for estimating the likelihood of ENI using routinely available clinical variables remain limited. We therefore sought to develop and internally evaluate a pragmatic prediction model for ENI after EVT in a real-world stroke cohort.

**Methods:**

We performed a single-centre retrospective cohort study at Shanxi Provincial People’s Hospital. A total of 314 EVT-treated patients were initially screened from hospital records. After preliminary data verification and assembly of the research database, 253 patients remained in the final study database available for variable-level assessment, of whom 185 with complete data on the primary outcome and prespecified key model variables were included in the primary complete-case analysis. ENI was defined as either a reduction in NIHSS score of at least 8 points from baseline to 1 week or an absolute 1-week NIHSS score of 1 or less. Candidate predictors included age, sex, baseline NIHSS, diabetes, cardioembolic aetiology, prior cerebrovascular disease, and door-to-puncture time (DPT). A multivariable logistic regression model was developed and translated into a simplified bedside score based on baseline NIHSS category and cardioembolic aetiology. Model performance was assessed using discrimination, calibration, Brier score, decision curve analysis, and bootstrap internal validation. Sensitivity analyses included an alternative ENI-4 definition, 48-hour neurological improvement as an alternative early outcome, alternative DPT thresholds, and multiple imputation for incomplete baseline covariates only.

**Results:**

Among the 185 patients in the primary analytical cohort, 53 (28.6%) achieved ENI. Baseline NIHSS was the dominant predictor of ENI in both univariable and multivariable analyses, whereas the additional contribution of other candidate predictors was modest. In the full model (Model 2), each 1-point increase in baseline NIHSS was associated with a 13% increase in the odds of ENI (adjusted OR 1.13, 95% CI 1.05–1.21; *p* < 0.001). The full model showed an apparent AUC of 0.706 and an optimism-corrected AUC of 0.657 after 1,000 bootstrap resamples; the corresponding Brier scores were 0.181 and 0.197. Bootstrap-corrected calibration suggested some overfitting (intercept − 0.335, slope 0.591). The simplified bedside score yielded an apparent and optimism-corrected AUC of 0.677, while the NIHSS-only model showed an apparent AUC of 0.673 and an optimism-corrected AUC of 0.674. Missing 1-week NIHSS was associated with higher baseline NIHSS, shorter length of stay, lower availability of 48-hour NIHSS, and worse discharge outcomes, suggesting that missing outcome data were unlikely to be completely random. Sensitivity analyses using alternative outcome definitions, alternative DPT thresholds, and multiple imputation for incomplete baseline covariates were broadly supportive of the primary findings, although some smaller-effect covariates were unstable in restricted subsets.

**Conclusions:**

In this single-centre real-world EVT cohort, baseline NIHSS emerged as the main predictor of early neurological improvement. A parsimonious model based on routinely available clinical variables showed only moderate discrimination, and the derived simplified bedside score may be useful for exploratory early risk stratification rather than as a stand-alone clinical decision tool. Given the substantial missingness in 1-week NIHSS, the possibility of selection bias, evidence of overfitting, and the absence of external validation, the model should be considered exploratory and requires independent validation before routine clinical use.

**Supplementary Information:**

The online version contains supplementary material available at 10.1186/s12883-026-04927-0.

## Introduction

Endovascular thrombectomy (EVT) has become the standard of care for selected patients with acute ischemic stroke due to large-vessel occlusion, after a series of landmark randomized controlled trials demonstrated substantial improvements in functional outcomes compared with best medical therapy alone [1–4]. These trials have reshaped acute stroke workflows and underscored the importance of rapid reperfusion. Nevertheless, outcomes after EVT remain heterogeneous, and a considerable proportion of patients fail to achieve functional independence despite technically successful recanalization [1–4]. In this context, early neurological improvement (ENI) has emerged as a pragmatic intermediate endpoint that reflects early treatment response and correlates with long-term prognosis.

ENI is typically defined according to changes in the National Institutes of Health Stroke Scale (NIHSS), most often as an improvement of ≥ 8–10 points or as achieving a NIHSS score of 0–1 within the first 24 h or by day 7 [5–8]. Several studies have shown that ENI after intravenous thrombolysis or EVT is strongly associated with favourable 90-day modified Rankin Scale (mRS) [9] scores and reduced mortality, and may serve as a surrogate endpoint for long-term outcomes in clinical research [5–8, 10]. For example, Wang et al. reported that ENI at day 1 following mechanical thrombectomy for distal medium-vessel occlusion stroke independently predicted good to excellent 3-month outcomes [10]. Similarly, large registry and cohort studies have highlighted that early neurological trajectories—encompassing both ENI and early neurological deterioration (END)—are key determinants of functional recovery after EVT [11, 12]. Collectively, these data underscore the clinical value of identifying, soon after thrombectomy, patients who are likely or unlikely to experience ENI.

Despite the growing interest in ENI, evidence on its prediction after EVT remains limited. Most prior work has focused on a restricted set of factors, such as baseline NIHSS, age, infarct core size, collateral status and degree of reperfusion, often examined in heterogeneous populations or within single vascular territories [11, 12]. A few groups have proposed multivariable models or nomograms for ENI or closely related early outcomes. Zhang et al. developed a nomogram to predict ENI in ischemic stroke patients treated with EVT, identifying baseline NIHSS and imaging markers as key predictors [13]. Wu et al. later constructed a nomogram to jointly predict 3-month unfavourable outcomes and ENI, further emphasizing the prognostic relevance of early neurological response [14]. More recently, machine learning–based nomograms have been reported for ENI after intravenous thrombolysis [11, 15]. However, many of these models are relatively complex, are not easily translated into simple bedside tools, and often lack comprehensive evaluation of calibration and clinical utility.

In parallel, there has been increasing attention on END and unexplained END after EVT, which are consistently associated with poor outcomes [11, 12, 16, 17]. Several nomograms have been proposed to predict END after mechanical thrombectomy, mainly in anterior circulation large-vessel occlusion [17, 18]. These studies highlight that early post-procedural neurological change—whether improvement or deterioration—reflects a complex interplay among baseline clinical status, vascular pathology and reperfusion quality. Nevertheless, ENI-specific prediction models after EVT remain relatively scarce, and even fewer have been developed and evaluated in contemporary real-world cohorts from Asian populations, where stroke etiologies, risk factor profiles and treatment workflows may differ from those in Western trials [11, 13–15]. Time metrics from hospital arrival to reperfusion represent another crucial dimension that may influence ENI. Workflow analyses from EVT trials and registries have shown that shorter door-to-puncture (DPT) or door-to-arterial puncture times are associated with higher rates of functional independence and better quality-of-life outcomes [19–22]. Quality improvement initiatives targeting DPT < 60 min have demonstrated that systematic process optimization can translate into improved clinical results [19, 21, 22]. However, the specific relationship between DPT and ENI after EVT is less well characterized, particularly in the ultra-late window (> 12 h from last known well), where patient selection and tissue viability are more heterogeneous. Existing data suggest that the impact of in-hospital delays may not be strictly linear across the entire time range, raising the possibility that non-linear modelling approaches, such as restricted cubic splines, could provide additional insight [11, 19–22]. 

From a methodological perspective, contemporary standards for clinical prediction research emphasize not only discrimination but also calibration and clinical usefulness. Decision curve analysis (DCA) provides a formal framework for quantifying the net benefit of prediction models across a range of threshold probabilities, and for comparing them with default “treat-all” and “treat-none” strategies [23, 24]. However, DCA has rarely been applied to ENI prediction after EVT, and few studies have integrated traditional regression modelling, simplified bedside scoring systems, nomograms and DCA within a single coherent framework.

Against this background, we sought to address several knowledge gaps using a real-world cohort of patients undergoing EVT in a high-volume centre. Our primary objective was to develop and internally evaluate a multivariable logistic regression model for predicting ENI after thrombectomy based on routinely available clinical variables. Building on this model, we aimed to derive a simplified bedside risk score and a corresponding nomogram to facilitate rapid risk stratification in everyday practice. A key secondary objective was to explore the independent and potentially non-linear association between DPT and ENI, with particular attention to patients treated in an ultra-late time window. To provide a comprehensive assessment, we examined model discrimination, Brier score, calibration and net benefit using receiver operating characteristic (ROC) analysis, calibration plots and DCA. Our study therefore seeks to deliver a pragmatic, clinically interpretable tool for early risk assessment after EVT, while also clarifying the extent to which in-hospital treatment delays influence early neurological recovery.

## Methods

### Study design and population

We conducted a single-centre retrospective cohort study at Shanxi Provincial People’s Hospital, including patients who underwent emergency endovascular thrombectomy (EVT) for acute ischemic stroke between 1 January 2020 and 31 December 2022. A total of 314 EVT-treated patients were initially identified from hospital records and screened for eligibility. Following preliminary data verification, removal of clearly ineligible or non-analyzable records, and assembly of the research database, 253 patients remained in the final study dataset available for variable-level assessment. Among these, 185 patients had complete data for the primary outcome and prespecified key model variables and were therefore included in the primary complete-case analysis.

Eligible patients were adults aged 18 years or older with acute ischemic stroke treated with EVT in either the anterior or posterior circulation. For inclusion in the primary complete-case analysis, patients were required to have an admission National Institutes of Health Stroke Scale (NIHSS) score, a follow-up 1-week NIHSS assessment, and complete information for the prespecified model variables. The study protocol was approved by the Ethics Committee of Shanxi Provincial People’s Hospital (approval No. 891). Given the retrospective design and the use of de-identified data, the requirement for written informed consent was waived. All study procedures complied with institutional requirements and the Declaration of Helsinki.

### Endpoints and definitions

The primary endpoint was early neurological improvement (ENI) after EVT. In the primary analysis, ENI was defined a priori as either a reduction of at least 8 points in NIHSS score from baseline to 1 week or an absolute 1-week NIHSS score of 1 or less. Patients who died before follow-up assessment were classified as not having ENI. Baseline NIHSS was defined as the first NIHSS score recorded on admission before EVT. The NIHSS is an established and validated measure of neurological deficit [25].

In prespecified sensitivity analyses, we also evaluated an alternative ENI definition based on a 4-point improvement (ENI-4), defined as either a reduction of at least 4 points in NIHSS score from baseline to 1 week or an absolute 1-week NIHSS score of 1 or less. In addition, we assessed 48-hour neurological improvement as an alternative early outcome, defined as either a reduction of at least 4 points in NIHSS score from baseline to 48 h or an absolute 48-hour NIHSS score of 1 or less.

### Candidate predictors

Candidate predictors were selected a priori on the basis of clinical relevance and previous literature on early neurological trajectories after reperfusion therapy. These included age, sex, baseline NIHSS, hypertension, diabetes, cardioembolic aetiology, prior cerebrovascular disease, and door-to-puncture time (DPT). DPT was defined as the interval between hospital arrival and arterial puncture for EVT.

Hypertension and diabetes were derived from the baseline combined vascular risk-factor history variable recorded in the study database. Patients were classified as having hypertension if this variable indicated hypertension alone or both hypertension and diabetes, and as having diabetes if it indicated diabetes alone or both conditions. Prior cerebrovascular disease was defined as any documented history of ischemic stroke, intracerebral hemorrhage, or transient ischemic attack. For the purposes of this study, cardioembolic aetiology was treated as a database-derived etiologic indicator based on the recorded baseline cardiac-source category, including atrial fibrillation and other documented cardiac embolic sources.

In the primary multivariable model, DPT was entered as a dichotomous variable (> 12 h vs. ≤ 12 h) to reflect ultra-late treatment. In additional sensitivity analyses, alternative DPT thresholds of > 6 h, > 9 h, and > 12 h were examined.

### Statistical analysis

#### Missing data handling

Missing data were assessed for all candidate predictors and outcome-defining variables in the final study database. Because the primary endpoint, early neurological improvement (ENI), depended directly on the 1-week NIHSS assessment, the primary analysis was based on a complete-case approach including only patients with observed outcome data and complete information on the prespecified model variables. Accordingly, of the 253 patients in the final study database, 185 were included in the primary complete-case analysis.

Missing 1-week NIHSS values were not imputed for the primary analysis because outcome missingness was considered likely to be related to routine clinical workflow, early discharge, inter-ward transfer, or incomplete reassessment at the prespecified timepoint, and was therefore unlikely to be completely random. To assess the potential for selection bias, patients included in the primary complete-case analysis were compared with those excluded from the analytical cohort within the final study database. In addition, characteristics associated with missing 1-week NIHSS assessment were examined to better characterize the likely mechanism of missingness.

As a sensitivity analysis for incomplete baseline covariates, multiple imputation by chained equations was performed among patients with observed primary outcome data. Outcome values were not imputed. Twenty imputed datasets were generated, and pooled estimates were obtained using Rubin’s rules. The extent and pattern of missing data, the comparison between included and excluded patients, characteristics associated with missing 1-week NIHSS assessment, and the multiple-imputation sensitivity analysis are presented in Supplementary Tables S3–S6.

#### Descriptive and univariable analyses

Continuous variables were inspected for distribution and are presented as mean ± standard deviation (SD) or median with interquartile range (IQR), as appropriate. Categorical variables are summarized as counts and percentages. Baseline characteristics were compared between patients with ENI and those without ENI using Welch’s t test for age, the Mann–Whitney U test for non-normally distributed continuous variables, and the χ² test or Fisher’s exact test for categorical variables, as appropriate. Baseline characteristics according to ENI status are summarized in (Table 1). 

**Table 1:** Baseline clinical characteristics according to early neurological improvement status. Values are presented as mean ± standard deviation, median (interquartile range), or number (percentage), as appropriate. ENI indicates early neurological improvement. Analytic cohort includes 185 patients (ENI, *n* = 53; no ENI, *n* = 132)

Variable	All patients (*n* = 185)	ENI (*n* = 53)	No ENI (*n* = 132)	*p*-value
Age, years	61.9 ± 13.2	62.3 ± 13.8	61.7 ± 13.0	0.785
Male sex, *n* (%)	125 (67.6%)	30 (56.6%)	95 (72.0%)	0.065
Pre-stroke mRS	0.0 (0.0–0.0)	0.0 (0.0–0.0)	0.0 (0.0–0.0)	0.382
Baseline NIHSS	14.0 (11.0–19.0)	18.0 (12.0–20.0)	13.0 (11.0–17.0)	< 0.001
Wake-up stroke, *n* (%)	48 (25.9%)	13 (24.5%)	35 (26.5%)	0.926
Hypertension, *n* (%)	101 (54.6%)	32 (60.4%)	69 (52.3%)	0.402
Diabetes, *n* (%)	27 (14.6%)	9 (17.0%)	18 (13.6%)	0.725
Prior cerebrovascular disease, *n* (%)	34 (18.4%)	10 (18.9%)	24 (18.2%)	1.000
Cardioembolic aetiology, *n* (%)	64 (34.6%)	22 (41.5%)	42 (31.8%)	0.279
Admission glucose, mmol/L	6.9 (6.0–8.4)	7.0 (6.2–8.3)	6.9 (6.0–8.4)	0.685
LDL cholesterol, mmol/L	2.7 (2.1–3.3)	2.6 (2.0–3.3)	2.8 (2.2–3.3)	0.389
HDL cholesterol, mmol/L	1.1 (0.9–1.3)	1.1 (0.9–1.2)	1.1 (0.9–1.3)	0.302
Heart rate on admission, bpm	83.2 ± 21.4	80.2 ± 18.6	84.4 ± 22.4	0.191
Door-to-puncture time, h	8.0 (5.0–11.0)	7.0 (4.5–10.0)	8.0 (5.0–11.0)	0.166

Univariable associations between each candidate predictor and ENI were evaluated using logistic regression. Results are reported as crude odds ratios (ORs) with 95% confidence intervals (CIs) and corresponding *p* values, as shown in (Table 2).

**Table 2:** Univariable associations between candidate predictors and early neurological improvement. Crude odds ratios (ORs) and 95% confidence intervals (CIs) from univariable logistic regression models with early neurological improvement (ENI) as the dependent variable. ENI indicates early neurological improvement; DPT, door-to-puncture time

Predictor	Crude OR (95% CI)	*p*-value
Age (per 10-year increase)	1.04 (0.81–1.32)	0.778
Male sex	0.51 (0.26–0.99)	0.045
Baseline NIHSS (per 1-point increase)	1.13 (1.06–1.21)	< 0.001
Diabetes	1.30 (0.54–3.10)	0.561
Cardioembolic disease	1.52 (0.79–2.94)	0.212
Prior cerebrovascular disease	1.05 (0.46–2.37)	0.913
Hypertension	1.39 (0.73–2.66)	0.318
Wake-up stroke	0.90 (0.43–1.88)	0.780
DPT (per 1-hour increase)	0.93 (0.85–1.02)	0.125
DPT > 12 h vs. ≤ 12 h	0.41 (0.11–1.45)	0.165

#### Multivariable model development

Multivariable logistic regression models were then fitted with ENI as the dependent variable. To preserve an adequate number of events per variable and reduce the risk of overfitting, we prespecified a parsimonious set of clinically relevant predictors rather than relying solely on data-driven variable selection. The core model (Model 1) included age (per 10-year increase), sex, baseline NIHSS (per 1-point increase), diabetes, cardioembolic aetiology, and prior cerebrovascular disease. A second model (Model 2) additionally incorporated DPT > 12 h to assess the incremental contribution of ultra-late treatment beyond the clinical core model.

For each model, adjusted odds ratios with 95% confidence intervals and *p* values were reported. The multivariable results of the primary analysis are summarized in (Table 3). The primary multivariable analyses were performed in the complete-case cohort. In the multiple-imputation sensitivity analysis, the same model structure was refitted after imputation of incomplete baseline covariates while preserving the observed primary outcome; the pooled results are presented in Supplementary Table S6. 

**Table 3:** Multivariable logistic regression models for early neurological improvement (Model 1: clinical core; Model 2: core + DPT > 12 h). Adjusted odds ratios (ORs) and 95% confidence intervals (CIs) from multivariable logistic regression models. Model 1 includes age, sex, baseline NIHSS, diabetes, cardioembolic disease, and prior cerebrovascular disease. Model 2 additionally includes door-to-puncture time (DPT) > 12 h. “—” indicates that the predictor was not included in the corresponding model. ENI indicates early neurological improvement; OR, odds ratio; CI, confidence interval; DPT, door-to-puncture time

Predictor	Adjusted OR (95% CI) – Model 1	*p*-value	Adjusted OR (95% CI) – Model 2	*p*-value
Age (per 10-year increase)	0.89 (0.68–1.16)	0.394	0.90 (0.68–1.18)	0.428
Male sex	0.52 (0.25–1.07)	0.077	0.51 (0.25–1.06)	0.072
Baseline NIHSS (per 1-point increase)	1.13 (1.05–1.21)	< 0.001	1.13 (1.05–1.21)	< 0.001
Diabetes	1.39 (0.54–3.55)	0.494	1.36 (0.53–3.49)	0.528
Cardioembolic disease	1.32 (0.63–2.75)	0.457	1.21 (0.57–2.55)	0.620
Prior cerebrovascular disease	1.02 (0.43–2.43)	0.960	1.08 (0.45–2.58)	0.863
DPT > 12 h	-	-	0.44 (0.12–1.66)	0.226

#### Simplified bedside score and nomogram

To enhance clinical usability, we derived a simplified bedside score consistent with (Table 4). Baseline NIHSS was categorized into three strata (≤ 10, 11–15, and ≥ 16) and assigned 0, 2, and 4 points, respectively. Cardioembolic aetiology contributed 1 point when present. The total score therefore ranged from 0 to 5 points and was further grouped into low (0–2), intermediate (3–4), and high (5) strata. The simplified score was then entered as a continuous predictor in a logistic regression model to estimate the predicted probability of ENI. In addition, a nomogram was constructed from the full multivariable model to provide an intuitive graphical tool for bedside estimation of ENI risk (Fig. 2). Given the modest incremental predictive value beyond baseline NIHSS alone, the simplified score was intended as an exploratory risk-stratification aid rather than a stand-alone clinical decision tool. 

**Table 4:** Simplified bedside risk score for early neurological improvement. Simplified point-based score derived from the multivariable model. Higher total scores indicate a higher predicted probability of early neurological improvement (ENI). The score uses only baseline NIHSS category and the presence of cardioembolic disease to facilitate bedside use

Predictor	Category	Points
Baseline NIHSS	≤10	0
	11–15	2
	≥16	4
Cardioembolic disease	Absent	0
	Present	1

Total score ranges and observed ENI rates in the derivation cohort		
Total score range	Risk category	Observed ENI rate
0–2	Low	15/92 (16.3%)
3–4	Intermediate	22/63 (34.9%)
5	High	16/30 (53.3%)

#### Model performance and internal validation

Model discrimination was quantified using the area under the receiver operating characteristic curve (AUC) with 95% confidence intervals (CIs). Overall prediction error was assessed using the Brier score, a proper scoring rule for probabilistic predictions [26, 27]. Calibration was evaluated by plotting observed versus predicted probabilities across deciles of predicted risk and by estimating the calibration intercept and slope from a logistic regression of the observed outcome on the logit of the predicted risk.

We compared the performance of three models: (1) an NIHSS-only model; (2) the full multivariable model (Model 2); and (3) the simplified bedside score. Receiver operating characteristic curves for these models are shown in (Fig. 3). Calibration plots for the full model and the simplified score are presented in (Fig. 4). Apparent performance metrics, including AUC, Brier score, calibration intercept, and calibration slope, are summarized in Supplementary Table S1. 

Internal validation of the primary model was performed using bootstrap resampling with 1,000 repetitions to quantify optimism and obtain optimism-corrected estimates of AUC, Brier score, calibration intercept, and calibration slope. The reporting and evaluation of the prediction model were guided by contemporary methodological recommendations [28, 29]. External validation was not performed.

#### Additional and sensitivity analyses

Several prespecified sensitivity analyses were performed to assess the robustness of the primary findings.

First, we evaluated an alternative ENI definition based on a 4-point improvement (ENI-4), defined as either a reduction of at least 4 points in NIHSS score from baseline to 1 week or an absolute 1-week NIHSS score of 1 or less. The corresponding sensitivity analysis is presented in Supplementary Table S7.

Second, we assessed 48-hour neurological improvement as an alternative early outcome, defined as either a reduction of at least 4 points in NIHSS score from baseline to 48 h or an absolute 48-hour NIHSS score of 1 or less. This analysis was restricted to patients with complete data for the alternative 48-hour outcome and the prespecified model variables, and the results are shown in Supplementary Table S8.

Third, to examine the stability of the DPT effect, we repeated the multivariable analysis using alternative dichotomous DPT thresholds of > 6 h, > 9 h, and > 12 h while retaining the original primary ENI definition. These threshold-based analyses are reported in Supplementary Table S9.

Fourth, restricted cubic spline models were used to explore the potential non-linear association between continuous DPT and ENI across the observed time range. Odds ratios for ENI across the DPT range were plotted using 6 h as the reference value, and 95% confidence intervals were displayed to illustrate estimation uncertainty, particularly in the ultra-late window (> 12 h) (Figure S2).

Fifth, we generated a forest plot of adjusted odds ratios from the main multivariable model to visually summarize the relative contribution of each predictor (Figure S1). Finally, decision curve analysis was performed to compare the net benefit of the full model, the simplified bedside score, a treat-all strategy, and a treat-none strategy across a range of clinically relevant threshold probabilities (Figure S3) [23, 24].

All analyses were conducted using R (R Foundation for Statistical Computing, Vienna, Austria) and Python (Python Software Foundation), with packages including statsmodels, scikit-learn, and rms. A two-sided* p* value < 0.05 was considered statistically significant.

## Results

### Study flow and analytical cohort

**Fig. 1:**
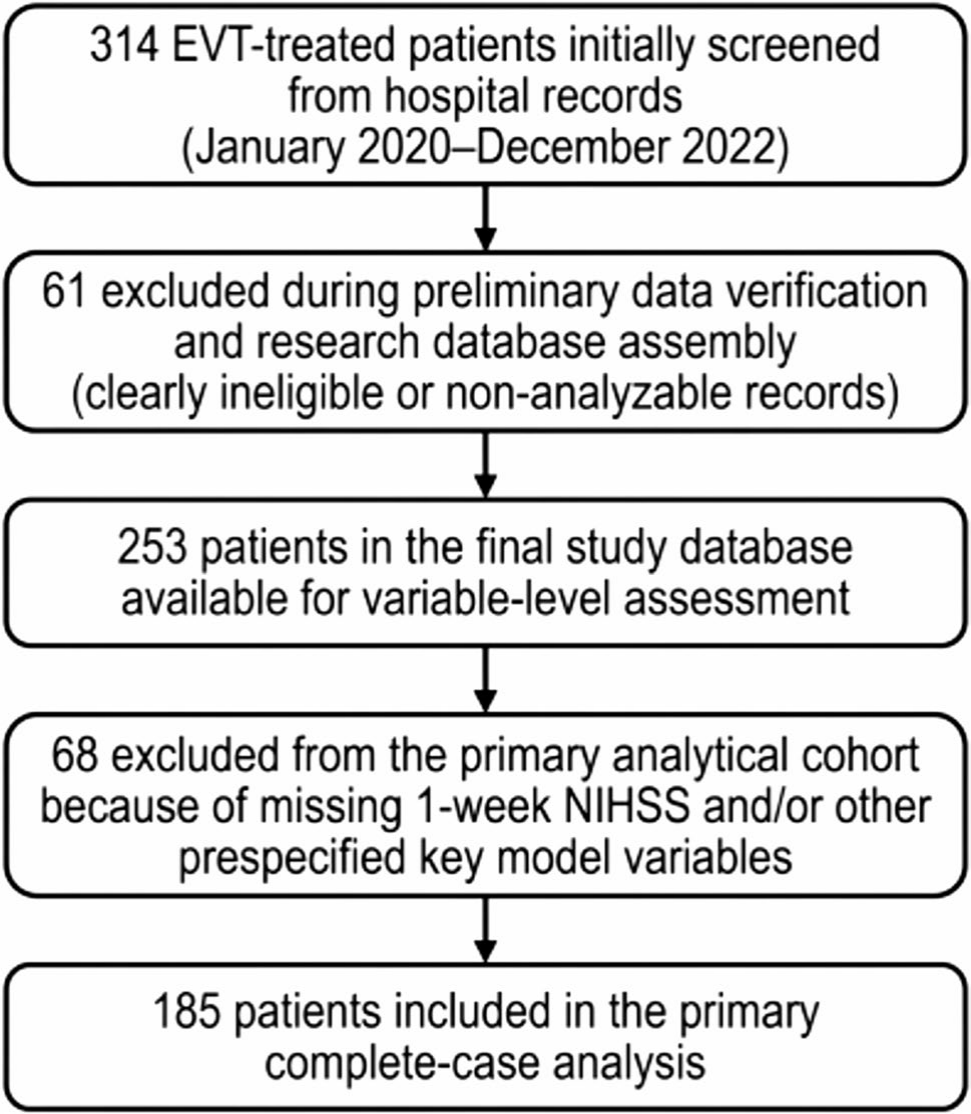
Flow chart of patient selection for the ENI prediction study among patients undergoing emergency mechanical thrombectomy at Shanxi Provincial People’s Hospital, January 2020–December 2022. A total of 314 EVT-treated patients were initially screened from hospital records. After preliminary data verification, exclusion of clearly ineligible or non-analyzable records, and assembly of the research database, 253 patients remained in the final study database available for variable-level assessment. Of these, 68 patients were excluded from the primary analytical cohort because of missing 1-week NIHSS and/or other prespecified key model variables, leaving 185 patients for the primary complete-case analysis. ENI, early neurological improvement; EVT, endovascular thrombectomy; NIHSS, National Institutes of Health Stroke Scale

A total of 314 EVT-treated patients were initially screened from hospital records during the study period. After preliminary data verification, exclusion of clearly ineligible or non-analyzable records, and assembly of the research database, 253 patients remained in the final study database available for variable-level assessment. Of these, 185 patients had complete data on the primary outcome and prespecified key model variables and were therefore included in the primary complete-case analysis. Within this analytical cohort, 53 patients (28.6%) met the primary definition of early neurological improvement (ENI), whereas 132 did not. The study flow is shown in (Fig. 1).

### Missing data overview and baseline characteristics

Missingness in the final study database was concentrated in follow-up neurological assessments and selected workflow-related variables rather than in most baseline demographic or clinical predictors. Specifically, 68 of 253 patients (26.9%) had missing 1-week NIHSS, 30 (11.9%) had missing 48-hour NIHSS, and 69 (27.3%) had missing DPT values. By contrast, age, sex, hypertension, diabetes, cardioembolic aetiology, prior cerebrovascular disease, and discharge outcome were essentially complete. The full pattern of missingness is summarized in Supplementary Table S3.

Baseline characteristics of the primary complete-case cohort according to ENI status are presented in (Table 1). Patients who achieved ENI had greater neurological severity at presentation, with a median baseline NIHSS of 18.0 (12.0–20.0) compared with 13.0 (11.0–17.0) in those without ENI (*p* < 0.001). Age was similar between groups (62.3 ± 13.8 vs. 61.7 ± 13.0 years, *p* = 0.785), as were the frequencies of hypertension, diabetes, and prior cerebrovascular disease. Cardioembolic aetiology was numerically more frequent in patients with ENI (41.5% vs. 31.8%), although this difference was not statistically significant. Median DPT was 7.0 (4.5–10.0) hours in the ENI group and 8.0 (5.0–11.0) hours in the non-ENI group (*p* = 0.166).

### Comparison between included and excluded patients

Patients included in the primary complete-case analysis were compared with those excluded from the analytical cohort within the final study database (Supplementary Table S4). Compared with excluded patients, those included in the analytical cohort had lower baseline NIHSS scores, longer hospital stays, lower admission glucose levels, markedly higher availability of 48-hour NIHSS assessments, and substantially more favorable discharge outcomes. Specifically, median baseline NIHSS was 14.0 (11.0–19.0) in the included group versus 18.0 (11.0–22.0) in the excluded group (*p* = 0.042). Median length of stay was 16.0 (13.0–23.0) days versus 4.0 (1.8–16.0) days (*p* < 0.001), and median admission glucose was 6.9 (6.0–8.4) mmol/L versus 8.5 (6.2–9.5) mmol/L (*p* = 0.014). Availability of 48-hour NIHSS was 100.0% in the analytical cohort and 55.9% in excluded patients (*p* < 0.001). Discharge outcomes also differed markedly overall (*p* < 0.001). In contrast, age, sex, hypertension, diabetes, cardioembolic aetiology, prior cerebrovascular disease, DPT, LDL, HDL, WBC, creatinine, and urea were broadly comparable between groups. Overall, these findings suggest that exclusion from the analytical cohort was driven primarily by follow-up completeness and hospitalization course rather than by major imbalance in baseline clinical characteristics.

### Factors associated with missing 1-week NIHSS assessment

To further characterize the mechanism of missingness, we compared patients with and without available 1-week NIHSS assessments (Supplementary Table S5). Missing 1-week NIHSS was associated with greater baseline neurological severity, shorter hospital stay, reduced availability of 48-hour NIHSS, and substantially worse discharge outcomes. Patients with missing 1-week NIHSS had a median baseline NIHSS of 18.0 (11.0–22.0), compared with 14.0 (11.0–19.0) in those with observed follow-up (*p* = 0.042). Median length of stay was markedly shorter in the missing group, at 4.0 (1.8–16.0) days versus 16.0 (13.0–23.0) days (*p* < 0.001). Only 55.9% of patients with missing 1-week NIHSS had a recorded 48-hour NIHSS, compared with 100.0% among those with observed 1-week follow-up (*p* < 0.001). Discharge outcomes also differed substantially: mortality was 48.5% in the missing group versus 9.7% in the observed group (*p* < 0.001), whereas favorable discharge outcome was much less common (44.1% vs. 90.3%, *p* < 0.001). Admission glucose was also higher in patients with missing follow-up, at 8.5 (6.2–9.5) mmol/L compared with 6.9 (6.0–8.4) mmol/L (*p* = 0.014). No clear differences were seen for age, sex, hypertension, diabetes, prior cerebrovascular disease, cardioembolic aetiology, or DPT. Taken together, these findings suggest that missing 1-week NIHSS was unlikely to be completely random and was more plausibly related to clinical severity and the course of hospitalization.

### Univariable associations

In univariable logistic regression analyses (Table 2), higher baseline NIHSS was strongly associated with increased odds of ENI (crude OR 1.13, 95% CI 1.06–1.21; *p* < 0.001). Male sex was associated with lower odds of ENI (OR 0.51, 95% CI 0.26–0.99; *p* = 0.045). Age was not significantly associated with ENI (OR 1.04 per 10-year increase, 95% CI 0.81–1.32; *p* = 0.778). Diabetes, cardioembolic aetiology, prior cerebrovascular disease, and hypertension were not significantly associated with ENI in univariable analyses. DPT > 12 h was directionally associated with lower odds of ENI (OR 0.41, 95% CI 0.11–1.45; *p* = 0.165), although the estimate was imprecise.

### Multivariable model

In the multivariable logistic regression analysis (Table 3), baseline NIHSS remained the dominant independent predictor of ENI. In Model 2, each 1-point increase in baseline NIHSS was associated with a 13% increase in the odds of ENI (adjusted OR 1.13, 95% CI 1.05–1.21; *p* < 0.001). Male sex retained a directionally negative association (adjusted OR 0.51, 95% CI 0.25–1.06; *p* = 0.072), although this did not reach conventional statistical significance. Diabetes (adjusted OR 1.36, 95% CI 0.53–3.49; *p* = 0.528), cardioembolic aetiology (adjusted OR 1.21, 95% CI 0.57–2.55; *p* = 0.620), and prior cerebrovascular disease (adjusted OR 1.08, 95% CI 0.45–2.58; *p* = 0.863) were not independently associated with ENI after adjustment. DPT > 12 h also remained directionally associated with lower odds of ENI (adjusted OR 0.44, 95% CI 0.12–1.66; *p* = 0.226), but the confidence interval was wide. Overall, the multivariable findings indicate that the model’s principal predictive signal was driven by baseline stroke severity, whereas the additional contribution of the remaining covariates was limited.

### Simplified bedside score and nomogram

**Fig. 2:**
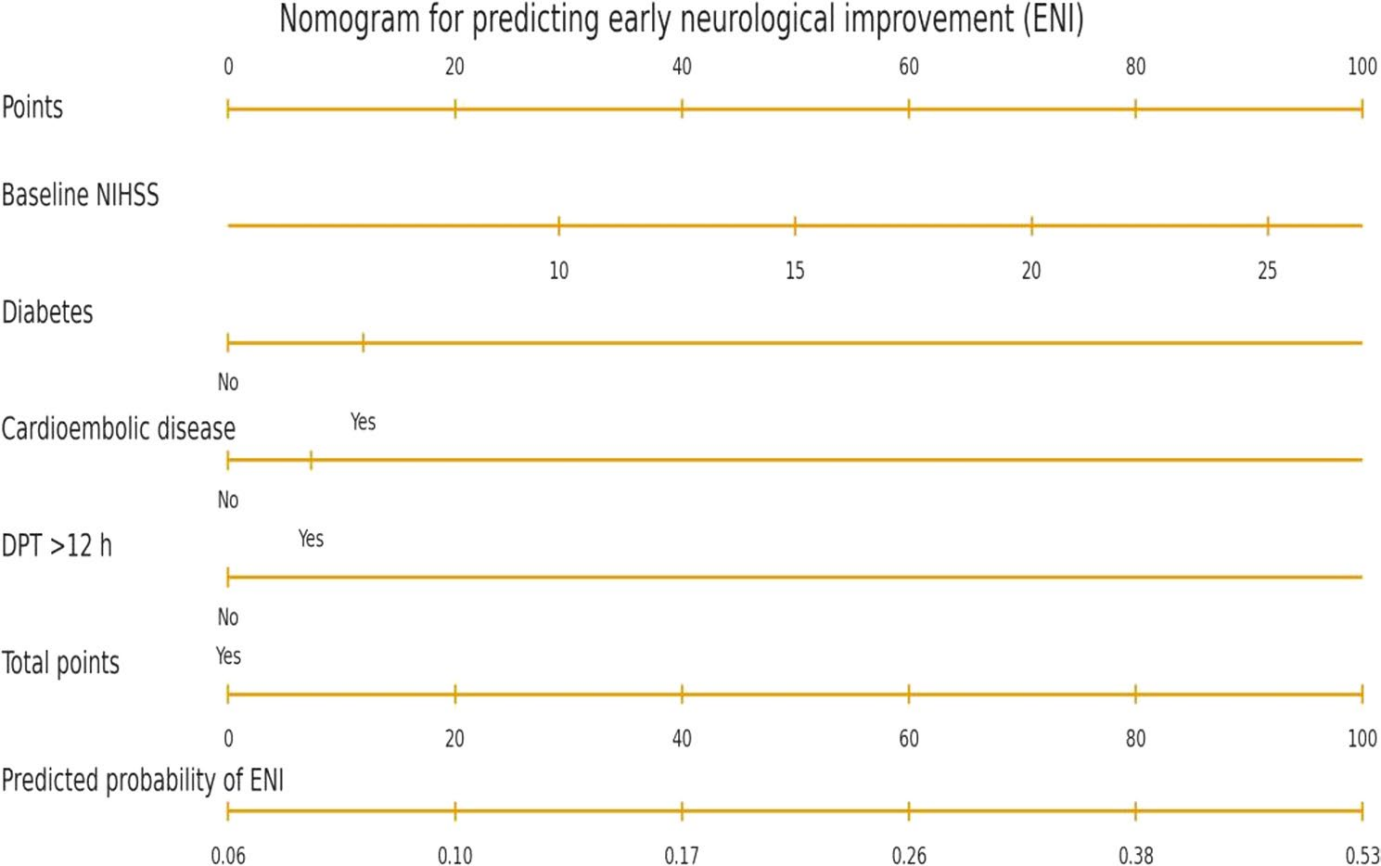
Nomogram for predicting early neurological improvement after thrombectomy

Based on the multivariable model, a simplified bedside score was constructed using baseline NIHSS category and cardioembolic aetiology (Table 4). Observed ENI rates increased across the predefined score strata: 15 of 92 patients (16.3%) in the low-risk group, 22 of 63 (34.9%) in the intermediate-risk group, and 16 of 30 (53.3%) in the high-risk group. The corresponding nomogram derived from the full multivariable model is shown in (Fig. 2) These findings suggest that the score may serve as an exploratory bedside risk-stratification tool, although most of its predictive information appears to be captured by baseline NIHSS.

### Model performance and internal validation

**Fig. 3:**
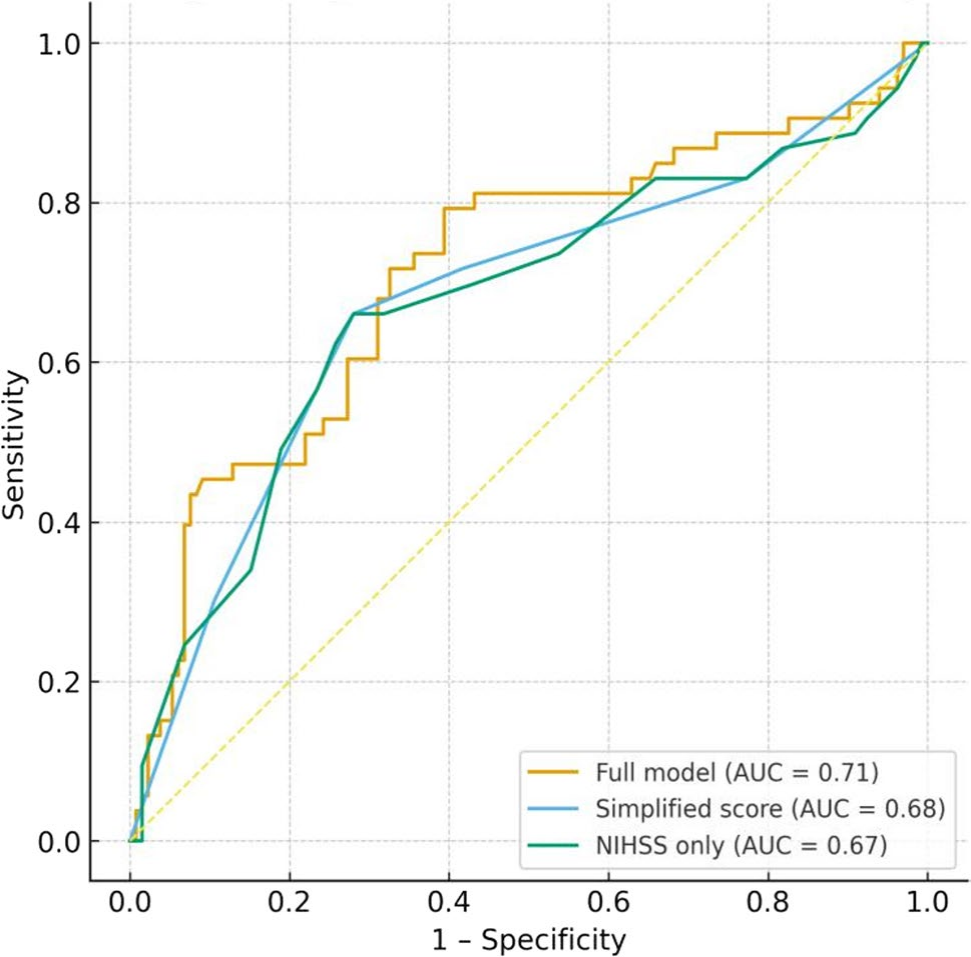
Receiver operating characteristic curves for ENI prediction models

**Fig. 4:**
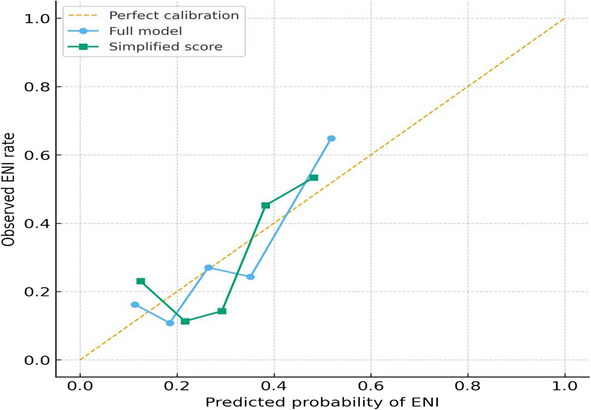
Calibration plots for the full model and simplified bedside score

The full multivariable model (Model 2) showed moderate apparent discrimination, with an AUC of 0.706. After bootstrap internal validation with 1,000 resamples, the optimism-corrected AUC was 0.657. The corresponding Brier score was 0.181 in the apparent analysis and 0.197 after optimism correction. Bootstrap-corrected calibration of the full model showed an intercept of − 0.335 and a slope of 0.591, indicating some degree of overfitting. By comparison, the simplified bedside score yielded an apparent and optimism-corrected AUC of 0.677, whereas the NIHSS-only model showed an apparent AUC of 0.673 and an optimism-corrected AUC of 0.674. Apparent and optimism-corrected performance metrics for all models are summarized in Supplementary Table S2. Receiver operating characteristic curves are shown in Fig. 3, and calibration plots are shown in (Fig. 4).

In decision curve analysis (Figure S3), both the full multivariable model and the simplified score provided greater net benefit than treat-all and treat-none strategies across a clinically relevant threshold range of approximately 0.20–0.50. However, the full model offered only modest incremental net benefit over the simplified score.

### Sensitivity analysis using an alternative ENI-4 definition

When ENI was alternatively defined as a reduction of at least 4 NIHSS points or a 1-week NIHSS score of 1 or less, 69 of 138 patients (50.0%) met the ENI-4 definition (Supplementary Table S7). In this restricted complete-case subset, baseline NIHSS remained directionally positively associated with early neurological improvement, with adjusted ORs of 1.06 (95% CI 0.99–1.13) in both Model 1 and Model 2. Age, male sex, and DPT > 12 h retained directions similar to those observed in the primary analysis. By contrast, some smaller-effect covariates, including diabetes and prior cerebrovascular disease, remained unstable and were estimated imprecisely in this restricted subset. Overall, the ENI-4 sensitivity analysis was compatible with the primary finding that baseline stroke severity was the dominant driver of early neurological response, although secondary associations were not reproduced exactly.

### Sensitivity analysis using 48-hour neurological improvement

In the sensitivity analysis using 48-hour neurological improvement as an alternative early outcome, 40 of 164 patients (24.4%) met the ENI-48 h definition (Supplementary Table S8). In this model, cardioembolic aetiology showed a stronger positive association than in the primary analysis and reached statistical significance in Model 1 (adjusted OR 2.24, 95% CI 1.03–4.89; *p* = 0.042), although the association attenuated in Model 2. DPT > 12 h showed a stronger negative crude association (OR 0.21, 95% CI 0.05–0.92; *p* = 0.039) and a borderline adjusted association in Model 2 (adjusted OR 0.23, 95% CI 0.05–1.02; *p* = 0.054). In contrast, baseline NIHSS did not show the same degree of association as in the primary 1-week model. These results were therefore broadly supportive but not identical to the primary analysis, suggesting that predictor importance may shift when the early neurological endpoint is assessed at 48 h rather than 1 week.

### Sensitivity analyses using alternative DPT thresholds

To assess the robustness of the DPT effect, we repeated the multivariable analyses using DPT thresholds of > 6 h, > 9 h, and > 12 h while retaining the original primary ENI definition (Supplementary Table S9). Across all three thresholds, longer DPT remained directionally associated with lower odds of ENI, with adjusted ORs of 0.80 (95% CI 0.36–1.77), 0.58 (95% CI 0.26–1.31), and 0.41 (95% CI 0.11–1.53), respectively. However, all confidence intervals were wide and crossed unity. These findings indicate that the direction of association was broadly consistent across alternative DPT thresholds, although the estimates remained imprecise.

Restricted cubic spline analysis of continuous DPT showed no strong overall non-linear association with ENI across the observed range (Figure S2). Odds ratios remained close to 1 across most of the DPT range, while estimates in the ultra-late window were accompanied by wide confidence intervals, reflecting limited precision in that range.

### Multiple-imputation sensitivity analysis

As a sensitivity analysis for incomplete baseline covariates, we performed multiple imputation by chained equations among patients with observed primary outcome data (Supplementary Table S6). The outcome-observed cohort comprised 185 patients, of whom 53 (28.6%) met the primary ENI definition; the corresponding complete-case model included 138 patients. In the imputed analysis, baseline NIHSS remained positively associated with ENI and became slightly stronger (MI OR 1.12, 95% CI 1.05–1.20; *p* = 0.001, versus complete-case OR 1.09, 95% CI 1.01–1.17; *p* = 0.031). The principal directions of association for age, male sex, cardioembolic aetiology, and DPT > 12 h were preserved, and the relative magnitude of the main associations was not materially altered. Overall, the multiple-imputation analysis did not change the primary conclusions and suggests that incomplete baseline covariates were unlikely to be the main driver of the observed results.

## Discussion

### Main findings

In this single-centre real-world EVT cohort, approximately one third of patients experienced early neurological improvement (ENI), and ENI again appeared to function as an early clinical marker linked to subsequent recovery, consistent with previous reports [7, 10]. The main finding of the present study is that baseline NIHSS was the dominant predictor of ENI, whereas other routinely available clinical variables—including diabetes, cardioembolic aetiology, prior cerebrovascular disease, and door-to-puncture time (DPT)—showed only modest and statistically imprecise associations.

We developed a multivariable logistic regression model with moderate discriminative performance and translated it into a simplified bedside score based on baseline NIHSS category and cardioembolic aetiology. The observed ENI rates across the predefined score strata (16.3%, 34.9%, and 53.3%) suggest that the score may offer a pragmatic framework for exploratory early risk stratification. At the same time, its performance gain over a NIHSS-only approach was limited, and the optimism-corrected calibration slope indicated some degree of overfitting. Decision curve analysis further suggested that both the full model and the simplified score performed better than treat-all and treat-none strategies across clinically relevant threshold probabilities, although the incremental net benefit of the more complex model was modest [23, 24, 28, 29].

Another important observation is that missing 1-week NIHSS was unlikely to be completely random. Patients without 1-week follow-up NIHSS had higher baseline NIHSS, shorter hospital stays, lower availability of 48-hour NIHSS, and substantially worse discharge outcomes. This pattern suggests that missingness was related to hospitalization course and clinical severity rather than to random data loss alone. This point is clinically and methodologically relevant, as it supports the use of a complete-case primary analysis while also underscoring the possibility of selection bias.

Sensitivity analyses were generally supportive of the main findings, although not identical in all respects. Across alternative outcome definitions and analytical strategies, baseline NIHSS remained the most stable predictor, whereas several smaller-effect covariates were directionally unstable in restricted subsets. Taken together, these findings indicate that early neurological response after EVT in this cohort was driven primarily by baseline stroke severity, while the independent contribution of other routinely collected clinical variables was comparatively limited.

### Comparison with previous studies

Our findings are consistent with previous studies showing that ENI, or closely related early neurological trajectories, is strongly associated with later functional outcome after reperfusion therapy [2, 7, 10]. Kobeissi et al. reported that broadly defined ENI after EVT was associated with higher rates of functional independence and lower mortality [7], and other observational studies have similarly emphasized the prognostic relevance of early post-treatment neurological change [2, 10]. In this context, the present results further support the clinical value of ENI as an intermediate recovery marker after thrombectomy.

Several groups have proposed models for predicting early neurological outcomes after EVT. Zhang et al. developed a multicentre nomogram incorporating age, glucose, ASPECTS, recanalisation status, and symptomatic intracranial haemorrhage, with stronger discrimination than that observed in the present study [30]. Li et al. found that advanced imaging variables, including CTP-ASPECTS, may improve prediction of early improvement after successful reperfusion [6]. More recently, Lai et al. examined ultra-early neurological improvement and similarly identified baseline stroke severity and imaging characteristics as major determinants of early recovery [6]. Compared with these studies, the present model was intentionally designed to rely on routinely available clinical variables only. This improves practical usability but probably comes at the cost of lower predic tive performance.

Our findings also align with the broader literature on early neurological deterioration and other early neurological trajectories after thrombectomy. Previous studies of early deterioration have emphasized the importance of baseline severity, infarct burden, vascular territory, and procedure-related factors [11, 13, 31]. Although the present study focused on ENI rather than deterioration, the overall pattern is similar: baseline neurological status appears to account for a large proportion of the prognostic signal, whereas the added contribution of individual clinical covariates is smaller and often estimated with limited precision.

With respect to workflow metrics, the broader EVT literature consistently supports the principle that shorter treatment delays are associated with better outcomes [2, 18, 32]. In our cohort, however, the independent association between DPT and ENI remained weak and imprecise after adjustment. This should not be interpreted as evidence against the “time is brain” concept. Rather, it more likely reflects the limited precision of DPT estimates in a modestly sized single-centre cohort, together with the possibility that ENI may be influenced more strongly by baseline tissue status than by relatively small in-hospital time differences once patients have already been selected for EVT [18, 32]. The consistency of the negative direction across alternative DPT thresholds supports this interpretation, even though the estimates remained unstable.

Compared with our earlier single-centre work, in which admission NIHSS and diabetes were associated with in-hospital neurological improvement, the present study extends that line of investigation methodologically. Here, we move beyond simple association analysis and adopt a broader prediction-model framework incorporating discrimination, calibration, internal validation, simplified scoring, and decision curve analysis, in line with current recommendations for model development and reporting [16, 23, 24, 28, 29, 33, 34].

### Clinical implications

From a practical perspective, the present findings suggest that baseline NIHSS remains the most informative routinely available bedside predictor of early neurological improvement after EVT. The simplified score may still be useful as an exploratory risk-stratification aid because it translates the main predictive information into an easily interpretable format. In this cohort, patients in the highest score stratum had an observed ENI rate of 53.3%, compared with 16.3% in the lowest stratum, indicating that even a simple model can identify clinically meaningful gradients of early recovery probability.

At the same time, the modest AUC, the limited gain over a NIHSS-only model, and the evidence of overfitting argue against presenting this tool as a stand-alone clinical decision instrument. Its most appropriate role is as an adjunct to clinical judgement—for example, to support exploratory early risk communication, to structure discussions about short-term neurological trajectory, or to inform future research hypotheses. It should not be used to deny EVT, justify withholding standard care, or replace more comprehensive prognostic assessment.

The sensitivity analyses also have clinical relevance. The fact that predictor importance shifted to some extent when the endpoint was defined at 48 h rather than 1 week suggests that early neurological recovery is time-dependent. Some predictors may be more relevant to very early postoperative change, whereas others may become more important over the first week. This supports a cautious interpretation of any single early-outcome model and highlights the need for validation across different clinically meaningful timepoints.

### Strengths and limitations

This study has several strengths. First, it is based on a real-world EVT cohort from a high-volume comprehensive stroke centre, which enhances its clinical relevance. Second, we used a structured prediction-model framework incorporating discrimination, calibration, Brier score, bootstrap internal validation, and decision curve analysis, consistent with established methodological guidance [23, 24, 28, 29]. Third, rather than limiting the analysis to statistical associations, we translated the main model into a simplified bedside score and nomogram, thereby improving interpretability. Fourth, and particularly relevant to this revision, we explicitly characterized the pattern and likely mechanism of missing follow-up data and supplemented the primary analysis with multiple sensitivity analyses, including alternative outcome definitions, alternative DPT thresholds, and multiple imputation of incomplete baseline covariates.

Several limitations should be acknowledged. Most importantly, the single-centre retrospective design limits generalisability and raises the possibility of selection bias. Missing 1-week NIHSS was substantial and appeared to be associated with clinical severity and hospitalization course. Although we did not impute outcome values in the primary analysis for sound methodological reasons, this does not remove the possibility that the complete-case cohort differed meaningfully from the broader EVT population represented in the final study database. Second, the sample size was modest, and the effective sample size became smaller in several sensitivity analyses restricted to alternative outcome definitions or complete variable sets. This likely contributed to the instability of some smaller-effect covariates and to the wide confidence intervals for variables such as DPT > 12 h.

Third, advanced imaging and procedural variables that could plausibly improve model performance—such as infarct core estimates, collateral status, infarct growth measures, or more detailed reperfusion characteristics—were not systematically available in a standardized form in this cohort. Their omission may partly explain why baseline NIHSS dominated the model and why overall discrimination remained only moderate [6, 31]. Fourth, although bootstrap internal validation was performed, the optimism-corrected calibration slope suggests some degree of overfitting, and external validation in an independent multicentre cohort is still needed. Finally, the primary endpoint was ENI rather than 90-day modified Rankin Scale. Although ENI is a clinically meaningful and previously studied early marker [7, 10], it does not fully capture delayed recovery, post-discharge complications, rehabilitation effects, or long-term functional outcome.

Taken together, these limitations support a cautious interpretation of the present model. It should be regarded as exploratory and hypothesis-generating rather than ready for routine clinical implementation. Future work should focus on external validation, refinement with richer imaging or procedural predictors, and evaluation of whether such a tool adds value beyond baseline NIHSS in more diverse EVT populations [2, 18, 32, 35].

## Conclusions

In this single-centre retrospective EVT cohort, baseline NIHSS emerged as the dominant predictor of early neurological improvement. A parsimonious model based on routinely available clinical variables showed only modest discrimination, and the derived simplified bedside score may be useful for exploratory early risk stratification rather than as a stand-alone clinical decision tool. Although sensitivity analyses using alternative outcome definitions, alternative DPT thresholds, and multiple imputation for incomplete baseline covariates were broadly supportive of the main findings, substantial missingness in 1-week NIHSS, possible selection bias, evidence of overfitting, and the absence of external validation remain important limitations. Accordingly, the model should be regarded as exploratory and requires independent validation before any routine clinical implementation.

## Supplementary Information

Supplementary Material 1.

## Data Availability

The de-identified patient-level dataset contains sensitive clinical information and cannot be publicly shared under institutional and legal requirements. Data may be made available from the Ethics Committee of Shanxi Provincial People’s Hospital (approval No. 891) upon reasonable request and with appropriate approvals and a data-use agreement.

## References

[1] Berkhemer OA, et al. A randomized trial of intraarterial treatment for acute ischemic stroke. N Engl J Med. 2015;372(1):11–20. 25517348 10.1056/NEJMoa1411587

[2] Goyal M, et al. Randomized assessment of rapid endovascular treatment of ischemic stroke. N Engl J Med. 2015;372(11):1019–30. 25671798 10.1056/NEJMoa1414905

[3] Saver JL, et al. Stent-retriever thrombectomy after intravenous t-PA vs. t-PA alone in stroke. N Engl J Med. 2015;372(24):2285–95. 25882376 10.1056/NEJMoa1415061

[4] Jovin TG, et al. Thrombectomy within 8 hours after symptom onset in ischemic stroke. N Engl J Med. 2015;372(24):2296–306. 25882510 10.1056/NEJMoa1503780

[5] Ong CT, et al. Early neurological improvement after intravenous tissue plasminogen activator infusion in patients with ischemic stroke aged 80 years or older. J Chin Med Assoc. 2014;77(4):179–83. 24657175 10.1016/j.jcma.2014.02.002

[6] Kurmann CC, et al. Association of the 24-Hour national institutes of health stroke scale after mechanical thrombectomy with early and long‐term survival. Volume 2. Stroke: Vascular and Interventional Neurology; 2022. p. e000244. 4. 10.1161/SVIN.121.000244 PMC12778784 41584762

[7] Kobeissi H, et al. Early neurological improvement as a predictor of outcomes after endovascular thrombectomy for stroke: a systematic review and meta-analysis. J Neurointerv Surg. 2023;15(6):547–51. 35636948 10.1136/neurintsurg-2022-019008

[8] Lai Y, et al. Identifying the predictors of ultra early neurological improvement and its role in functional outcome after endovascular thrombectomy in acute ischemic stroke. Front Neurol. 2025;16:1492013. 39958613 10.3389/fneur.2025.1492013 PMC11825449

[9] van Swieten JC, et al. Interobserver agreement for the assessment of handicap in stroke patients. Stroke. 1988;19(5):604–7. 3363593 10.1161/01.str.19.5.604

[10] Wang M, et al. Early neurological improvement predicts clinical outcome after thrombectomy for distal medium vessel occlusions. Front Neurol. 2022;13:809066. 35321507 10.3389/fneur.2022.809066 PMC8936066

[11] Huang Z, et al. Infarct growth rate predicts early neurological improvement in ischemic stroke after endovascular thrombectomy. Brain Sci. 2025;15(3):303. 10.3390/brainsci15030303. 10.3390/brainsci15030303 PMC11940323 40149824

[12] Dai Z, et al. Predictors of unexplained early neurological deterioration after thrombectomy for posterior circulation infarction: a reanalysis of the BASILAR study. J Neurosurg. 2024;141(6):1705–11. 38905713 10.3171/2024.4.JNS232632

[13] Zhang X, et al. Nomogram predicting early neurological improvement in ischaemic stroke patients treated with endovascular thrombectomy. Eur J Neurol. 2021;28(1):152–60. 32897575 10.1111/ene.14510

[14] Wu Y, et al. Nomogram-based prediction of 3-month unfavorable outcome and early neurological deterioration after endovascular thrombectomy in acute ischemic stroke. Ther Clin Risk Manag. 2025;21:239–56. 40035072 10.2147/TCRM.S505897 PMC11874981

[15] LvB-H, et al. A machine learning-based predictive nomogram for early neurological improvement after thrombolysis in acute ischemic stroke. Front Neurol. 2025;16:1662498. 10.3389/fneur.2025.1662498. 10.3389/fneur.2025.1662498 PMC12646877 41312351

[16] Wu K, et al. A nomogram predicts early neurological deterioration after mechanical thrombectomy in patients with ischemic stroke. Front Neurol. 2023;14:1255476. 37799278 10.3389/fneur.2023.1255476 PMC10548384

[17] Luo B, et al. A novel nomogram predicting early neurological deterioration after intravenous thrombolysis for acute ischemic stroke. Heliyon. 2024;10(1):e23341. 38163222 10.1016/j.heliyon.2023.e23341 PMC10757001

[18] Menon BK, et al. Analysis of workflow and time to treatment on thrombectomy outcome in the Endovascular Treatment for Small Core and Proximal Occlusion Ischemic Stroke (ESCAPE) randomized, controlled trial. Circulation. 2016;133(23):2279–86. 27076599 10.1161/CIRCULATIONAHA.115.019983

[19] Joundi RA, et al. Time from hospital arrival until endovascular thrombectomy and patient-reported outcomes in acute ischemic stroke. JAMA Neurol. 2024;81(7):752–61. 38829660 10.1001/jamaneurol.2024.1562 PMC11148789

[20] Chung HI, et al. Delayed door to puncture time during off-duty hours is associated with unfavorable outcomes after mechanical thrombectomy in the early window of acute ischemic stroke. BMC Neurol. 2024;24(1):357. 39342130 10.1186/s12883-024-03874-y PMC11438392

[21] Gilbert F, et al. Door-to-puncture time in ischemic stroke with large vessel occlusion in France: Patient and hospital factors. Rev Neurol (Paris). 2025;181(6):556–62. 40328544 10.1016/j.neurol.2025.03.013

[22] Liu Z, et al. Reducing door-to-puncture times for mechanical thrombectomy in a large tertiary hospital. Neurol Clin Pract. 2024;14(5):e200325. 38939047 10.1212/CPJ.0000000000200325 PMC11201277

[23] Vickers AJ, Elkin EB. Decision curve analysis: a novel method for evaluating prediction models. Med Decis Mak. 2006;26(6):565–74. 10.1177/0272989X06295361 PMC2577036 17099194

[24] Vickers AJ, van Calster B, Steyerberg EW. A simple, step-by-step guide to interpreting decision curve analysis. Diagn Progn Res. 2019;3:18. 31592444 10.1186/s41512-019-0064-7 PMC6777022

[25] Brott T, et al. Measurements of acute cerebral infarction: a clinical examination scale. Stroke. 1989;20(7):864–70. 2749846 10.1161/01.str.20.7.864

[26] Kattan MW, Cowen ME, editors. Encyclopedia of Medical Decision Making. Thousand Oaks: SAGE Publications; 2009. ISBN: 9781412953726.

[27] Steyerberg EW, et al. Assessing the performance of prediction models: a framework for traditional and novel measures. Epidemiology. 2010;21(1):128–38. 20010215 10.1097/EDE.0b013e3181c30fb2 PMC3575184

[28] Collins GS, et al. Transparent Reporting of a multivariable prediction model for Individual Prognosis or Diagnosis (TRIPOD): the TRIPOD statement. Ann Intern Med. 2015;162(1):55–63. 25560714 10.7326/M14-0697

[29] Moons KG, et al. Transparent Reporting of a multivariable prediction model for Individual Prognosis or Diagnosis (TRIPOD): explanation and elaboration. Ann Intern Med. 2015;162(1):W1–73. 25560730 10.7326/M14-0698

[30] Lai Y, et al. Predictors of failure of early neurological improvement in early time window following endovascular thrombectomy: a multi-center study. Front Neurol. 2023;14:1227825. 37780716 10.3389/fneur.2023.1227825 PMC10538528

[31] Li Y, et al. Predictors of early neurological improvement in patients with anterior large vessel occlusion and successful reperfusion following endovascular thrombectomy-does CT perfusion imaging matter? Clin Neuroradiol. 2022;32(3):839–47. 35244728 10.1007/s00062-022-01147-0 PMC9424155

[32] Weyland CS, et al. Predictors for failure of early neurological improvement after successful thrombectomy in the anterior circulation. Stroke. 2021;52(4):1291–8. 33626903 10.1161/STROKEAHA.120.030519

[33] Zhang Z, et al. Decision curve analysis: a technical note. Ann Transl Med. 2018;6(15):308. 30211196 10.21037/atm.2018.07.02 PMC6123195

[34] Zhou J, et al. Development and Validation of a nomogram for predicting the 6-year risk of cognitive impairment among Chinese older adults. J Am Med Dir Assoc. 2020;21(6):864–e8716. 32507532 10.1016/j.jamda.2020.03.032 PMC7299771

[35] Desai S, et al. P-027Ultra-early functional improvement after stroke thrombectomy – predictors and implications. J NeuroInterventional Surg. 2021;13(Sup1):1.

