# Supplementary material for: Development of a simple clinical score to predict early neurological improvement after mechanical thrombectomy: a single-centre cohort study
Source: BMC Neurol. 2026 May 9;26:427. doi: 10.1186/s12883-026-04927-0 (PMC13325569; doi:10.1186/s12883-026-04927-0)
Supplement: Supplementary file 1 — Supplementary Material 1. [file 12883_2026_4927_MOESM1_ESM.docx]

Table S1. Sensitivity analyses of the multivariable model for early neurological improvement

| **Predictor** | **Model 1 All patients (standard ENI)* Adjusted OR (95% CI)** | **Model 1 p-value** | **Model 2 Anterior circulation only Adjusted OR (95% CI)** | **Model 2 p-value** | **Model 3 ENI defined as NIHSS decrease ≥8 only Adjusted OR (95% CI)** | **Model 3 p-value** |
| --- | --- | --- | --- | --- | --- | --- |
| **NIHSS_baseline (per 1-point increase)** | 1.13 (1.05–1.21) | <0.001 | 1.17 (1.07–1.28) | <0.001 | 1.23 (1.14–1.34) | <0.001 |
| **Diabetes** | 1.41 (0.56–3.54) | 0.468 | 1.55 (0.47–5.08) | 0.467 | 2.00 (0.72–5.53) | 0.181 |
| **Cardioembolic disease** | 1.23 (0.61–2.48) | 0.558 | 1.25 (0.55–2.85) | 0.594 | 1.32 (0.61–2.86) | 0.480 |
| **DPT >12 h** | 0.45 (0.12–1.66) | 0.229 | 0.75 (0.19–2.90) | 0.674 | 0.35 (0.07–1.70) | 0.193 |

*Standard ENI definition: NIHSS decrease ≥8 points or NIHSS ≤1 at 1 week.

**Supplementary Table S2. Performance metrics with bootstrap internal validation**

Notes: App. = apparent performance on the development cohort. Corr. = optimism-corrected performance estimated by 1,000 bootstrap resamples. Calibration intercept and slope are obtained by regressing the observed outcome on the model logit(predicted risk).Supplementary Table S2 reports apparent performance and optimism-corrected estimates obtained from 1,000 bootstrap resamples (AUC, Brier score, calibration intercept and slope) for the full model, simplified score, and NIHSS-only model.

| **Model** | **AUC (App.)** | **AUC (Corr.)** | **Brier (App.)** | **Brier (Corr.)** | **Cal intercept (App.)** | **Cal intercept (Corr.)** | **Cal slope (App.)** | **Cal slope (Corr.)** | **Bootstraps (n)** |
| --- | --- | --- | --- | --- | --- | --- | --- | --- | --- |
| Full model (Model 2) | 0.706 | 0.657 | 0.181 | 0.197 | -0.001 | -0.335 | 1.000 | 0.591 | 1000 |
| Simplified bedside score (score logistic) | 0.677 | 0.677 | 0.186 | 0.191 | 0.000 | -0.108 | 1.000 | 0.893 | 1000 |
| NIHSS-only | 0.673 | 0.674 | 0.187 | 0.191 | -0.000 | 0.170 | 1.000 | 1.190 | 1000 |

**Supplementary Table S3**

Extent and pattern of missing data in the study database

**Table note.** *Missingness was concentrated in follow-up neurological assessment variables, particularly 1-week NIHSS, whereas most baseline demographic and clinical predictors had low levels of missing data.*

| **Variable** | **Total N** | **Number missing** | **% missing** |
| --- | --- | --- | --- |
| Age | 253 | 0 | 0.0 |
| Sex | 253 | 0 | 0.0 |
| Baseline NIHSS | 253 | 1 | 0.4 |
| 48h NIHSS | 253 | 30 | 11.9 |
| 1-week NIHSS | 253 | 68 | 26.9 |
| Hypertension | 253 | 0 | 0.0 |
| Diabetes | 253 | 0 | 0.0 |
| Cardioembolic aetiology | 253 | 0 | 0.0 |
| Prior CVD | 253 | 0 | 0.0 |
| DPT | 253 | 69 | 27.3 |
| Glucose | 253 | 32 | 12.6 |
| LDL | 253 | 22 | 8.7 |
| HDL | 253 | 34 | 13.4 |
| WBC | 253 | 2 | 0.8 |
| Creatinine | 253 | 5 | 2.0 |
| Urea | 253 | 4 | 1.6 |
| Length of stay | 253 | 3 | 1.2 |
| Discharge outcome | 253 | 0 | 0.0 |

**Abbreviations:** NIHSS, National Institutes of Health Stroke Scale; CVD, cerebrovascular disease; DPT, door-to-puncture time; LDL, low-density lipoprotein; HDL, high-density lipoprotein; WBC, white blood cell count.

Percentages were calculated using the total number of patients in the available study database (N = 253) as the denominator.

**Supplementary Table S4**

Comparison between patients included in the primary complete-case analysis and those excluded from the analytical cohort within the final study database

| **Variable** | **Included in primary complete-case analysis (n=185)** | **Excluded from analytical cohort (n=68)** | **P value** |
| --- | --- | --- | --- |
| Age, years | 61.9 ± 13.2 | 60.9 ± 12.8 | 0.598 |
| Male sex, n (%) | 125 (67.6%) | 44 (64.7%) | 0.781 |
| Baseline NIHSS | 14.0 (11.0–19.0) | 18.0 (11.0–22.0) | 0.042 |
| Hypertension, n (%) | 101 (54.6%) | 39 (57.4%) | 0.804 |
| Diabetes, n (%) | 27 (14.6%) | 12 (17.6%) | 0.689 |
| Cardioembolic aetiology, n (%) | 64 (34.6%) | 18 (26.5%) | 0.284 |
| Prior cerebrovascular disease, n (%) | 34 (18.4%) | 13 (19.1%) | 1.000 |
| Door-to-puncture time, h | 8.0 (5.0–11.0) | 8.0 (5.6–11.8) | 0.474 |
| Length of stay, days | 16.0 (13.0–23.0) | 4.0 (1.8–16.0) | <0.001 |
| Admission glucose, mmol/L | 6.9 (6.0–8.4) | 8.5 (6.2–9.5) | 0.014 |
| LDL cholesterol, mmol/L | 2.7 (2.1–3.3) | 2.7 (2.0–3.3) | 0.842 |
| HDL cholesterol, mmol/L | 1.1 (0.9–1.3) | 1.1 (0.9–1.3) | 0.749 |
| WBC, ×10^9/L | 9.2 (7.1–12.0) | 9.9 (7.7–12.3) | 0.176 |
| Creatinine, μmol/L | 63.4 (54.1–74.5) | 69.2 (56.7–82.9) | 0.081 |
| Urea, mmol/L | 5.3 (4.3–6.3) | 5.2 (3.7–6.6) | 0.672 |
| 48h NIHSS available, n (%) | 185 (100.0%) | 38 (55.9%) | <0.001 |
| Discharge outcome, n (%) |  |  | <0.001 |
| Improved | 167 (90.3%) | 30 (44.1%) |  |
| Death | 18 (9.7%) | 33 (48.5%) |  |
| Self-discharge/unknown | 0 (0.0%) | 5 (7.4%) |  |

Abbreviations: NIHSS, National Institutes of Health Stroke Scale; DPT, door-to-puncture time; CVD, cerebrovascular disease; LDL, low-density lipoprotein; HDL, high-density lipoprotein; WBC, white blood cell count. This corrected table aligns the coding of hypertension and diabetes with the combined history variable used in the main manuscript table. The table summarizes differences between patients included in the primary complete-case analysis (n = 185) and those excluded from the analytical cohort (n = 68) within the final study database available for variable-level assessment (N = 253), rather than in the initially screened hospital cohort (N = 314). Continuous variables are presented as mean ± standard deviation or median (interquartile range), as appropriate; categorical variables are presented as number (%). P values were calculated using Welch’s t test for age, Mann–Whitney U tests for non-normally distributed continuous variables, Fisher’s exact or chi-square tests for binary categorical variables as appropriate, and a chi-square test for the overall discharge outcome distribution.

**Supplementary Table S5**

Characteristics associated with missing 1-week NIHSS assessment

| **Variable** | **1-week NIHSS available (n=185)** | **1-week NIHSS missing (n=68)** | **P value** |
| --- | --- | --- | --- |
| Age, years | 61.9 ± 13.2 | 60.9 ± 12.8 | 0.598 |
| Male sex, n (%) | 125 (67.6%) | 44 (64.7%) | 0.781 |
| Baseline NIHSS | 14.0 (11.0–19.0) | 18.0 (11.0–22.0) | 0.042 |
| Hypertension, n (%) | 101 (54.6%) | 39 (57.4%) | 0.804 |
| Diabetes, n (%) | 27 (14.6%) | 12 (17.6%) | 0.689 |
| Prior cerebrovascular disease, n (%) | 34 (18.4%) | 13 (19.1%) | 1.000 |
| Cardioembolic aetiology, n (%) | 64 (34.6%) | 18 (26.5%) | 0.284 |
| Admission glucose, mmol/L | 6.9 (6.0–8.4) | 8.5 (6.2–9.5) | 0.014 |
| Door-to-puncture time, h | 8.0 (5.0–11.0) | 8.0 (5.6–11.8) | 0.474 |
| Length of stay, days | 16.0 (13.0–23.0) | 4.0 (1.8–16.0) | <0.001 |
| 48h NIHSS available, n (%) | 185 (100.0%) | 38 (55.9%) | <0.001 |
| Discharge outcome: improved, n (%) | 167 (90.3%) | 30 (44.1%) | <0.001 |
| Discharge outcome: died, n (%) | 18 (9.7%) | 33 (48.5%) | <0.001 |
| Discharge outcome: self-discharge/other, n (%) | 0 (0.0%) | 5 (7.4%) | 0.001 |

Footnote: This corrected table summarizes characteristics associated with missing 1-week NIHSS assessment within the final study database (N = 253), consistent with the study flow of 314 initially screened, 253 available in the final study database, and 185 in the primary complete-case model. Hypertension and diabetes were coded using the combined baseline history variable to match the main manuscript Table 1. Length of stay was parsed from the original hospitalization-duration text field. Continuous variables are presented as mean ± standard deviation or median (interquartile range), as appropriate; categorical variables are presented as n (%). P values were calculated using Welch’s t test for age, Mann–Whitney U tests for non-normally distributed continuous variables, and χ² test or Fisher’s exact test for categorical variables, as appropriate. NIHSS, National Institutes of Health Stroke Scale; DPT, door-to-puncture time; CVD, cerebrovascular disease.

**Supplementary Table S6**

Multiple imputation sensitivity analysis for incomplete baseline covariates

| **Predictor** | **Complete-case OR (95% CI)** | **P value** | **MI OR (95% CI)** | **P value** |
| --- | --- | --- | --- | --- |
| Age (per 10-year increase) | 0.94 (0.68–1.31) | 0.729 | 0.90 (0.69–1.18) | 0.453 |
| Male sex | 0.55 (0.24–1.23) | 0.144 | 0.50 (0.24–1.05) | 0.066 |
| Baseline NIHSS (per 1-point increase) | 1.09 (1.01–1.17) | 0.031 | 1.12 (1.05–1.20) | 0.001 |
| Diabetes | 0.37 (0.04–3.35) | 0.376 | 0.39 (0.04–3.48) | 0.402 |
| Cardioembolic aetiology | 1.19 (0.52–2.73) | 0.685 | 1.20 (0.56–2.55) | 0.637 |
| Prior cerebrovascular disease | 0.79 (0.29–2.12) | 0.634 | 1.01 (0.42–2.40) | 0.989 |
| DPT >12 h | 0.41 (0.11–1.53) | 0.184 | 0.54 (0.14–2.00) | 0.354 |

Footnote: The primary outcome retained the original study definition of early neurological improvement (ENI), defined as a decrease in NIHSS of ≥8 points from baseline to 1 week or an absolute 1-week NIHSS score ≤1. Multiple imputation by chained equations was applied to incomplete baseline covariates only, while outcome values were not imputed. The outcome-observed cohort comprised 185 patients, of whom 53 (28.6%) met the primary ENI definition; the corresponding complete-case model included 138 patients. Twenty imputed datasets were generated and pooled using Rubin’s rules. This supplementary analysis is aligned with the overall study flow of 314 initially screened, 253 available in the final study database, and 185 in the primary complete-case model. OR, odds ratio; CI, confidence interval; NIHSS, National Institutes of Health Stroke Scale.

**Supplementary Table S7**

Sensitivity analysis using an alternative ENI definition based on 4-point improvement

| **Predictor** | **Crude OR (95% CI)** | **P value** | **Adjusted OR (95% CI) – Model 1** | **P value** | **Adjusted OR (95% CI) – Model 2** | **P value** |
| --- | --- | --- | --- | --- | --- | --- |
| Age (per 10-year increase) | 0.96 (0.73–1.26) | 0.766 | 0.91 (0.68–1.22) | 0.535 | 0.92 (0.68–1.23) | 0.555 |
| Male sex | 0.83 (0.42–1.66) | 0.597 | 0.76 (0.36–1.59) | 0.465 | 0.76 (0.36–1.60) | 0.474 |
| Baseline NIHSS (per 1-point increase) | 1.06 (0.99–1.13) | 0.092 | 1.06 (0.99–1.13) | 0.107 | 1.06 (0.99–1.13) | 0.110 |
| Diabetes | 0.38 (0.07–2.04) | 0.260 | 0.41 (0.08–2.29) | 0.313 | 0.40 (0.07–2.23) | 0.296 |
| Cardioembolic aetiology | 1.14 (0.56–2.29) | 0.721 | 1.09 (0.52–2.28) | 0.829 | 1.05 (0.49–2.24) | 0.895 |
| Prior cerebrovascular disease | 0.91 (0.39–2.12) | 0.830 | 0.87 (0.37–2.05) | 0.742 | 0.87 (0.37–2.07) | 0.757 |
| DPT >12 h | 0.79 (0.31–2.05) | 0.629 | — | — | 0.80 (0.30–2.14) | 0.657 |

Footnote: ENI-4 was defined as a decrease in NIHSS of ≥4 points from baseline to 1 week, or an absolute 1-week NIHSS score ≤1. Model 1 includes age (per 10-year increase), sex, baseline NIHSS, diabetes, cardioembolic aetiology, and prior cerebrovascular disease. Model 2 additionally includes door-to-puncture time (DPT) >12 h. The analysis was performed in the complete-case cohort with non-missing values for the outcome and prespecified model variables (n = 138); 69 patients (50.0%) met the ENI-4 definition. This supplementary analysis is aligned with the study flow of 314 initially screened, 253 available in the final study database, and 185 in the primary complete-case model. OR, odds ratio; CI, confidence interval; NIHSS, National Institutes of Health Stroke Scale; DPT, door-to-puncture time.

**Supplementary Table S8**

Sensitivity analysis using 48-hour neurological improvement as an alternative early outcome

| **Predictor** | **Crude OR (95% CI)** | **P value** | **Adjusted OR (95% CI) – Model 1** | **P value** | **Adjusted OR (95% CI) – Model 2** | **P value** |
| --- | --- | --- | --- | --- | --- | --- |
| Age (per 10-year increase) | 0.92 (0.69–1.22) | 0.557 | 0.83 (0.61–1.12) | 0.220 | 0.83 (0.61–1.13) | 0.230 |
| Male sex | 0.85 (0.41–1.77) | 0.673 | 0.84 (0.39–1.83) | 0.669 | 0.82 (0.37–1.81) | 0.627 |
| Baseline NIHSS (per 1-point increase) | 1.02 (0.96–1.10) | 0.483 | 1.02 (0.95–1.10) | 0.508 | 1.02 (0.95–1.10) | 0.553 |
| Diabetes | 0.37 (0.05–3.07) | 0.358 | 0.38 (0.04–3.23) | 0.373 | 0.31 (0.04–2.70) | 0.292 |
| Cardioembolic aetiology | 2.08 (1.00–4.34) | 0.051 | 2.24 (1.03–4.89) | 0.042 | 1.95 (0.88–4.30) | 0.099 |
| Prior cerebrovascular disease | 0.80 (0.32–2.01) | 0.635 | 0.82 (0.32–2.11) | 0.680 | 0.87 (0.33–2.28) | 0.780 |
| DPT >12 h | 0.21 (0.05–0.92) | 0.039 | — | — | 0.23 (0.05–1.02) | 0.054 |

Footnote: In this sensitivity analysis, 48-hour neurological improvement was defined as a decrease in NIHSS of ≥4 points from baseline to 48 hours, or an absolute 48-hour NIHSS score ≤1. Model 1 includes age (per 10-year increase), sex, baseline NIHSS, diabetes, cardioembolic aetiology, and prior cerebrovascular disease. Model 2 additionally includes door-to-puncture time (DPT) >12 h. The analysis was restricted to patients with complete data for the alternative 48-hour outcome and all prespecified model variables (n = 164); 40 patients (24.4%) met the ENI-48h definition. This supplementary analysis is aligned with the overall study flow of 314 initially screened, 253 available in the final study database, and 185 in the primary complete-case model, but uses a restricted complete-case subset for the alternative outcome analysis. OR, odds ratio; CI, confidence interval; NIHSS, National Institutes of Health Stroke Scale; DPT, door-to-puncture time.

**Supplementary Table S9**

Sensitivity analyses using alternative door-to-puncture time thresholds

| **DPT threshold comparison** | **Complete-case n** | **ENI events, n (%)** | **Crude OR (95% CI)** | **P value** | **Adjusted OR (95% CI)** | **P value** |
| --- | --- | --- | --- | --- | --- | --- |
| DPT >6 h vs ≤6 h | 138 | 41 (29.7%) | 0.69 (0.33–1.45) | 0.328 | 0.80 (0.36–1.77) | 0.577 |
| DPT >9 h vs ≤9 h | 138 | 41 (29.7%) | 0.59 (0.27–1.29) | 0.187 | 0.58 (0.26–1.31) | 0.187 |
| DPT >12 h vs ≤12 h | 138 | 41 (29.7%) | 0.37 (0.10–1.35) | 0.131 | 0.41 (0.11–1.53) | 0.184 |

Footnote: Sensitivity analyses were performed using alternative dichotomous door-to-puncture time (DPT) thresholds of 6, 9, and 12 hours. The primary outcome retained the original study definition of early neurological improvement (ENI), defined as a decrease in NIHSS of ≥8 points from baseline to 1 week or an absolute 1-week NIHSS score ≤1. Adjusted models included age (per 10-year increase), sex, baseline NIHSS, diabetes, cardioembolic aetiology, prior cerebrovascular disease, and the corresponding DPT threshold indicator. Each threshold analysis was restricted to patients with complete data for the outcome and all prespecified model variables; >6 h subset: n=138, ENI events=41; >9 h subset: n=138, ENI events=41; >12 h subset: n=138, ENI events=41. This supplementary analysis is aligned with the overall study flow of 314 initially screened, 253 available in the final study database, and 185 in the primary complete-case model, but uses threshold-specific restricted complete-case subsets. OR, odds ratio; CI, confidence interval; NIHSS, National Institutes of Health Stroke Scale.
